# Adapting to Change

**DOI:** 10.1371/journal.pbio.1001606

**Published:** 2013-07-09

**Authors:** Jonathan M. Chase

**Affiliations:** Freelance Science Writer, Saint Louis, Missouri, United States of America

Biologists typically use the climatic conditions in which species currently live to project the likelihood that those climates will be available following a period of climate change, and these almost always predict dramatically high rates of species extinctions and biodiversity loss in future climates. In many instances, such predictions are warranted. Polar bears (*Ursus maritimus*), for example, are projected to be at high extinction risk as a result of the global warming-induced loss of arctic sea ice on which they depend for hunting their prey. For other species, however, the effect of climate change on extinction risk is less obvious. Because species can adapt to changing environments through both phenotypic plasticity (changes in behavior, physiology, and/or morphology) and micro-evolutionary adaptation, many can persist and even thrive in the face of changing climates.

**Figure:**
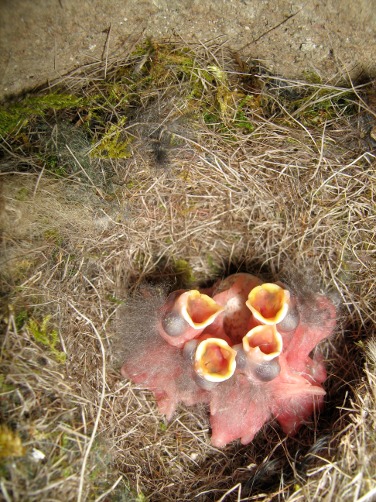
Newly hatched great tit *Parus major* nestlings. Data from a long-term study of this species are used to explore the adaptive significance of phenotypic plasticity in timing of breeding, and the importance of phenotypic plasticity in adjustment to climate change. Image credit: Simon Evans, Uppsala University.

A recent theoretical framework suggests that the likelihood of a species adapting to changing climates to avoid extinction results from the interaction between phenotypic plasticity and micro-evolutionary change, as well as environmental (rate of climate change) and demographic (life history) variables. While this theory's utility lies in its ability to predict which populations will escape extinction due to climate change through adaptation, the data necessary to make these predictions require explicit knowledge of both phenotypic plasticity and the strengths of selection in response to variable climates, which are rarely available at the appropriate scale and thus has not been attempted. That is, until the analyses by Vedder and colleagues published in this issue of *PLOS Biology*.

Vedder and colleagues used data from a population of great tits (*Parus major*) at Wytham Woods, a moderately sized forested site near Oxford University that is one of the most intensively studied bird populations ever. This population has been monitored in the same way each spring since 1960, using more than 1,000 nestboxes on site. A key piece of data collected throughout this time period is the timing in which birds lay their eggs relative to spring temperatures, and in particular, how well they track the timing of peak caterpillar abundances, a critical food these birds need to successfully rear their young. These birds now lay their eggs on average two weeks earlier than they did 50 years ago, primarily as a result of phenotypic plasticity in response to a concurrent shift in the timing of peak caterpillar numbers.

By using the relationship between egg-laying date and temperature among hundreds of individuals breeding each year, Veddar and colleagues calculated the parameters necessary to project population-level changes in the egg-laying date and the likelihood of population persistence or extinction. Specifically, they estimated the genetic variance of egg-laying date, the strength of selection on laying date (the numbers of offspring of a parent returning the next year), how that selection varied with temperature (and the timing of peak caterpillar numbers), and the phenotypic plasticity in egg-laying date with respect to temperature. From this parameterized model, they predicted that this great tit population could adapt and persist in an environment that was warming up to 0.5°C per year; much higher than even the most dramatic predicted warming from climate models (0.03°C per year). Even when they included uncertainty in the parameter estimates, the population had less than one-tenth of one percent chance of going extinct with the highest projected climate warming. Importantly, the incorporation of phenotypic plasticity was critical for these projections; without plasticity allowing the birds to adjust the timing of their egg-laying, the population had a 60% chance of going extinct.

Although their study provided a compelling case study for the need to include both plasticity and microevolutionary dynamics to project extinctions due to climate change, Veddar and colleagues wanted to take their simulation models beyond great tits in Wytham Woods to see if they could learn something more general about adaptation to climate change. First, they varied the life history parameters in their model, including generation time and population growth rate, finding that species with slower life histories (long generations and low population growth) are less likely to be able to track changing environments and are more at risk of extinction than shorter-lived species with higher population growth. Second, they varied the degree of genetic variability and strengths of selection, again concluding that this great tit population and other species like it are likely to be able to adapt to even the most pessimistic climate change scenarios owing to the interaction between plasticity and microevolutionary adaptation.

The great tits of Wytham Woods are able to adapt in the face of climate change through a combination of phenotypic plasticity and microevolutionary response in egg-laying date, allowing them to track changes in caterpillar densities. Other species might also be able to track changing climates through the interplay between plasticity and microevolution, although their ability to do so will depend critically on their life histories; shorter-lived species will adapt more readily than those that are longer-lived.

**Vedder O, Bouwhuis S, Sheldon BC (2013) Quantitative Assessment of the Importance of Phenotypic Plasticity in Adaptation to Climate Change in Wild Bird Populations. doi:10.1371/journal.pbio.1001605**

